# The global, regional, and national burden of foreign bodies from 1990 to 2019: a systematic analysis of the global burden of disease study 2019

**DOI:** 10.1186/s12889-024-17838-x

**Published:** 2024-01-31

**Authors:** Jiangshan Zhan, Yuxia Du, Jin Wu, Fuping Lai, Renjie Song, Yu Wang, Zhihui Xie, Yuanlan Lu

**Affiliations:** 1 Department of Emergency, Affiliated Hospital of Zunyi Medical University, 149 Dalian Road, Zunyi, China; 2 School of Medicine, Zhejiang University, Hangzhou, China; 3 Department of Hyperbaric Oxygen, Affiliated Hospital of Zunyi Medical University, Zunyi, China; 4 Shanxi Bethune Hospital, Shanxi Academy of Medical Sciences, Tongji Shanxi Hospital, Third Hospital of Shanxi Medical University, Taiyuan, China; 5 Outpatient Department, Affiliated Hospital of Zunyi Medical University, Zunyi, China

**Keywords:** Foreign body, Global burden of disease study, Trends, Incidence, Death

## Abstract

**Background:**

Foreign bodies (FBs) are a common emergency in medical institutions, that can occur in any area and among people of any age, which are common public health problems. Understanding the epidemiological characteristics of FBs is crucial for their prevention and control. The purpose of this study was to analyze the epidemiological characteristics of FBs worldwide through the data from the Global Burden of Disease Study 2019 (GBD 2019).

**Methods:**

We obtained data from the GBD 2019, which is an important public database to understand the disease burden of FBs. Joinpoint was used to analyze temporal trends in the incidence and death trends of FBs, which is widely used to study the long-term temporal trend of the burden of diseases. SaTScan was used to detect spatial-temporal clusters of pulmonary aspiration and foreign body in the airway (PAFBA), which is based on a Poisson model, scanning the number of people and diseases in the study area to obtain the spatial-temporal clusters of diseases.

**Results:**

Globally, the age-standardized incidence rate (ASIR) and the age-standardized death rate (ASDR) of FBs in 2019 were 869.23/100,000 (679.92/100,000-1120.69/100,000) and 1.55/100,000 (1.41/100,000-1.67/100,000), respectively. The ASIR and ASDR showed downtrends with average annual percent changes (AAPCs) of -0.31% and − 1.47% from 1990 to 2019. Of note, the ASIR showed an uptrend during 2010–2019, especially in high, high-middle, and middle SDI regions. Stratified analysis by age group showed that ASIR increased in each age group in recent years. From 1990 to 2019, the ASDR in the over-70 age group showed an uptrend worldwide, especially in high and high-middle SDI regions. In different types of FBs, the ASDR of PAFBA was the highest. The death burden of PAFBA was mainly clustered in 82 countries during 1993–2007, such as Canada, Cuba, and Mexico.

**Conclusion:**

The most important goal is to improve public awareness and emergency knowledge of FBs through publicity methods, such as the internet or offline activities, and to improve laws and regulations. Additionally, different age groups need different targeted measures, such as strengthening the care of children, caring for elderly individuals, improving necessary monitoring programs and reporting systems, conducting effective hazard assessments, and publicity and education activities.

**Supplementary Information:**

The online version contains supplementary material available at 10.1186/s12889-024-17838-x.

## Introduction

FBs are a common clinical emergency, that involves people of all ages, especially children under the age of 5 and older adults over 80 years old [1]. Globally, foreign bodies (FBs), including intraocular foreign bodies (IOFBs), pulmonary aspiration and foreign bodies in the airway (PAFBA), and FBs in other body parts, are any object or substance that unintentionally penetrates the body or its cavities. Among them, IOFBs are extraneous materials or substances that unintentionally damage tissue and can stay in the orbital structure or eye, causing different degrees of damage to the eyes According to the position and nature of IOFBs, they can be manifested as eye pain, tears, eyelid spasms, and even inflammatory reactions [2, 3]. Foreign body ingestions (FoBIs) are the unintentional or intentional swallowing or inhalation of substances or objects that are not naturally present in the body, which is a common clinical emergency. According to the size, entry site, and residence time of FB, there may be no clinical manifestations or choking, wheezing, dyspnea, or abnormal breath sounds in the trachea [4, 5]. FBs in other body parts, such as FB in the esophagus, can be clinically manifested as FB obstruction, dysphagia, pain, nausea, vomiting and other gastrointestinal reactions, fever, and other different manifestations [2, 6]. The risk factors of FBs in different parts are different. For example, the risk factors of IOFBs are age, trauma, and bad living habits such as rubbing eyes, staring at mobile phones for a long time, and working in an environment with flying debris and dust for a long time. The risk factors of PAFBA are age, laughing and talking while eating, etc. The risk factors for FBs in the digestive tract are age, mental disorder, and malignant tumor [2, 6, 7].

Various complications may occur due to anatomical location, intelligence level, treatment time, and national economic and cultural differences, and even lead to death in severe cases [8, 9]. Among them, the most common complications of FBs in the eye are blindness or loss of eyeballs and unilateral visual impairment [2, 10]. The long-term complications of PAFBA include atelectasis, pneumonia, and bronchiectasis, and the most serious complications are apnea caused by hypoxia, ischemic hypoxic encephalopathy, and death [11]. FBs in other parts of the body, such as FBs in the digestive tract, may cause gastrointestinal bleeding, perforation, and ulcers, and its most serious complications are sepsis and septic shock leading to intestinal necrosis [12].

The Global Burden of Disease Study (GBD) estimates reveal that the overall death rate from FBs is approximately 1.5 per 100,000 population, and FBs are the 63rd leading cause of death and the 16th leading cause of injury worldwide, implying that FBs are a global health concern [13, 14]. Previous studies reported that in 2019, the incidence of Foreign body aspiration (FBA) among children under 5 years old was 109.6/100,000. Especially in many European countries (such as Italy, Netherlands, Iceland, etc.) showed a high incidence rate, with the incidence exceeding 350/100,000 [15]. In addition, the global incident number of IOFB increased from 35.79 million in 1990 to 46.63 million in 2019, an increase of 30.29%. Among them, the incidence rate in Oceania was low in 1990 and 2019, while South Asia was the region with the largest number of cases in 2019, especially in India [2]. It is reported that the death rate in 17 countries in Europe, Asia, Africa, and America is between 0% and 8.3% [16]. In 2018, in the United States, FBs were the third leading cause of accidental deaths and the second leading cause in Japan in 2019 [17]. FBs have brought a great burden to public health events.

Currently, Most FB cases can be prevented by effective measures. These include raising public awareness and emergency knowledge through publicity, improving laws and regulations, striving to strengthen the care of children, caring for elderly individuals, and other measures. Differences between regions may be related to the rapid development of social and economic conditions, further improvement of basic health care preventive measures, and regional differences in culture. In recent years, the current research mainly focuses on how to use new technologies and methods to identify and deal with FBs more effectively, reduce the impact of FBs on the body, and explore the potential impact and complications of FBs on the body; With the development of medical technology, minimally invasive surgery, nanotechnology, etc. are gradually concerned about FBs [9, 10]. However, there are few epidemiological studies in this field, and only a few studies have explained the epidemiological trend of IOFBs and trachea, which cannot comprehensively evaluate the global incidence and death trend of FBs. Therefore, to understand the current burden and prevalence trends of FBs by region, SDI, age, and sex, and to assess the influence of these factors on FBs burden and trends. In this study, we aim to analyze the burden and long-term trend of FBs worldwide from 1990 to 2019 using the data from the Global Disease Burden Study in 2019 (GBD 2019), and to conduct subgroup analysis according to region, age, and sex. To provide a more comprehensive perspective for the development of global and regional targeted FB prevention interventions and health policies.

## Methods

### Data sources and manipulation

Our data come from the GBD 2019 database, which comprehensively covers epidemiological models and health system assessments. In the process of data processing, data quality evaluation is carried out and data are continuously processed to enhance comparability, they constantly review the response rate, geographical coverage of the survey, or the integrity of the census. Evaluate the data of cohort studies and trials from multiple dimensions, including confounding risk, generalizability, reporting bias, and publication bias [18]. The GBD 2019 data were provided by the Global Health Data Exchange of the Institute for Health Metrics and Evaluation (IHME) at Washington University; the GBD 2019 provides comprehensive and standardized health assessments for 369 diseases and injuries, 282 causes of death, and 87 risk factors across 204 countries [13, 14]. The GBD 2019 categorizes 204 countries into 5 regions based on SDI and 21 regions based on their epidemiological similarity and geographical proximity. The age-standardized incidence rate (ASIR) and the age-standardized death rate (ASDR) by age, sex, location, and SDI for the global burden of FBs were directly downloaded from the website (http://ghdx.healthdata.org/gbd-results-tool), a catalog of global health and population data that relies on the GBD 2019 and includes FBs in eyes, FBs in other body parts and PAFBA. The data are provided to the public free of charge, and this study did not require ethical approval or informed consent. R software (version 4.2.1) was used for statistical analyses and visualizations.

### Sociodemographic index and study variables

The SDI, developed by the IHME at Washington University, is an essential tool to assess the development level of a country or region and compare it with those of other countries. This SDI is a composite indicator that measures human development by combining three factors: per capita income, education level in years, and total fertility rate among people younger than 25 years, using a weighted average. The resulting value is then ranked comprehensively. It is helpful to evaluate the development level of regions regarding FB incidents. The SDI of the Global Health Data Exchange (GHDx) categorizes countries and regions into five groups (high, high-middle, middle, low-middle, and low SDI), expressed on a scale of 0 to 1. The closer the value is to 1, the higher the development level [19].

The following were used to present study results from 1990 to 2019: cases (incidence and deaths), changes in the number of cases (incidence and deaths), age-standardized rates (ASIR and ASDR) (per 100,000 population), average annual percent changes (AAPCs), different types of FBs by age group and sex, 21 regions, 5 SDI regions, 204 countries and 95% uncertainty intervals (UIs). The GBD 2019 global standard population was used to calculate the number of incidences and deaths, as well as the ASIR, ASDR, and 95% UI. The detailed methods were explained in the GBD 2019 report, on the official website, and in previous studies [14, 20].

### Analysis of the joinpoint regression model

Joinpoint regression analysis, also known as piecewise regression, broken-line regression, or multi-phase regression, was first proposed by Kim in 2000. Based on the time characteristics of disease distribution, the model constructs piecewise regression and carries out trend fitting and optimization on the data points of each segment. Joinpoint regression analysis is a commonly used statistical method to analyze the time trend of diseases. In recent years, Joinpoint regression analysis has been widely used to study the long-term time trend of the burden of diseases. The advantage of using the joinpoint regression model is that there is no strict requirement on whether there is a trend in the data series itself. The data distribution type can be the normal distribution, exponential distribution, or Poisson distribution. However, when carrying out disease trend analysis, the research period should not be too short, generally at least five years in a row, and it is best to reach a period of more than 10 years in a row. If the time is too short, the research results will be greatly affected by factors such as the integrity and quality of the monitored data, and it is difficult to obtain objective trend change characteristics [21–24].

In our research, we used Joinpoint software (version 4.9.0.0) to perform a Joinpoint regression analysis on the temporal trend changes in the ASR of FBs worldwide from 1990 to 2019 and calculated the APC and AAPC and their 95% confidence interval (95% CI) for the entire study period. The Monte Carlo substitution method was employed to test the statistical significance over periods. Moreover, the AAPC was utilized to characterize the temporal trend of the ASR of FBs. The value of the AAPC represents the annual percent change (increase, decrease, or no change). By comparing the AAPC with 0, we determined whether the changing trend of different periods was statistically significant. When the AAPC > 0, the ASR increases with time, and when the AAPC < 0, the ASR decreases with time. If the AAPC is 0, it indicates that the ASR is stable. The significance test level was set at *P* < 0.05. This was a secondary analysis of GBD data, and there were no additional detailed covariate data to control for deviations. Therefore, the Joinpoint regression model may have been biased in evaluating the trend of the disease burden over time.

### Spatial-temporal aggregation analysis

SaTScan uses spatial, temporal, and spatial-temporal scan statistics to analyze spatial, temporal, and spatial-temporal data and can detect the spatial-temporal clustering of certain diseases. It was used to detect and evaluate the spatial-temporal clustering of PAFBA. A circular or deformable space-time scan statistic with variable size was generated on the map, the Poisson distribution model was used, and the maximum spatial clustering scale was set to 50% risk population. Finally, the log-likelihood ratio (LLR) was calculated by comparing the expected value within the window with the observed value, and the clustering areas were classified according to the LLR value. P values were tested based on the Monte Carlo hypothesis, and *P* < 0.05 was considered statistically significant.

## Results

### Global trends

**Fig. 1:**
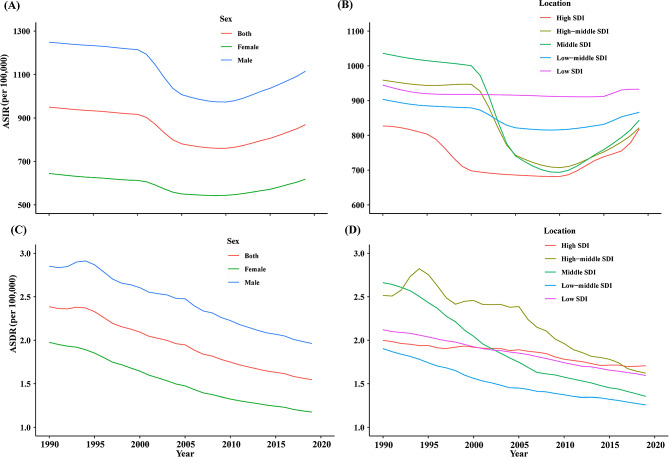
Temporal trends of ASIR and ASDR of foreign bodies globally and in different SDI regions from 1990 to 2019. (**A**) Global ASIR, (**B**) ASIR in SDI regions, (**C**) Global ASDR, (**D**) ASDR in SDI regions. ASIR: age-standardized incidence rate; ASDR: age-standardized death rate

On a global scale, the total incidence number of cases of FBs increased from 52,525,883 (95% UI 39,572,502–69,035,234) in 1990 to 66,522,520 (95% UI 51,681,454–86,328,194) in 2019. From 1990 to 2019, the ASIR showed a downward trend (AAPC = -0.31%, *P* < 0.05). Of note, it showed an upward trend between 2010 and 2019, which was divided into two stages, 2010–2015 (APC = 1.20%, *P* < 0.05) and 2015–2019 (APC = 1.91%, *P* < 0.05). (Fig. 1; Table 1, Supplementary material 1). This may be closely related to social and economic development, changes in people’s lifestyles and eating habits, and the development of medical diagnostic technology. The number of deaths from FBs decreased from 124,633 (95% UI 115,712–135,620) to 114,413 (95% UI 104,996–123,667). The ASDR showed a downward trend (AAPC = -1.47%, *P* < 0.05) (Fig. 1; Table 2).

**Table 1:** The incidence numbers and ASIR of foreign body in 1990 and 2019 with AAPCs over the 30 years

	1990			2019			
	Incidence number(95%UI)	ASIR (95%UI)		Incidence number(95%UI)	ASIR (95%UI)		AAPC, %(95%CI)
Global	52,525,883 (39,572,502–69,035,234)	950.05 (718.1-1261.59)		66,522,520 (51,681,454–86,328,194)	869.23 (679.92-1120.69)		-0.31*(-0.37, -0.26)
Gender							
Male	34,826,149 (25,497,703–46,664,809)	1248.89 (915.38-1673.96)		43,408,573 (32,858,330–57,276,213)	1115.36 (851.43-1460.77)		-0.41*(-0.49, -0.33)
Female	17,699,734 (14,114,352–22,561,924)	644.53 (512.45-822.34)		23,113,947 (18,531,029–29,212,350)	618.6 (498.92-775.12)		-0.14*(-0.23, -0.05)
SDI rank							
High SDI	6,507,826 (5,219,773–8,258,418)	827.14 (667.71-1037.69)		7,333,658 (5,902,922–9,172,380)	818.45 (659.71-1029.07)		-0.06 (-0.13, 0.01)
High-middle SDI	11,157,083 (8,207,821–15,196,513)	959.01 (715.24-1288.35)		11,287,820 (8,150,221–15,665,650)	821.78 (612.25-1099.25)		-0.53*(-0.64, -0.43)
Middle-SDI	18,276,857 (12,767,949–25,460,938)	1036.24 (728.99-1457.19)		20,374,554 (14,814,836–27,777,166)	843.57 (624.71-1126.66)		-0.74*(-0.89, -0.59)
Low-middle SDI	10,871,382 (8,630,992–13,758,539)	903.61 (716.55-1149.5)		15,729,298 (12,477,354–20,058,086)	866.34 (694.77-1098.12)		-0.14*(-0.19, -0.09)
Low SDI	5,692,713 (4,621,762–7,097,836)	944.45 (773.49-1174.22)		11,767,367 (9,469,806–14,719,665)	932.8 (762.02-1159.78)		-0.03 (-0.06, 0)
GBD regions							
Andean Latin America	498,791 (412,419–601,337)	1095.9 (916.31-1324.82)		584,490 (473,297–724,453)	906.76 (737.18-1126.46)		-0.65*(-0.66, -0.63)
Australasia	251,488 (203,855–308,472)	1299.44 (1049.18-1597.6)		324,226 (269,480–392,373)	1291.95 (1050.1-1586.41)		-0.02*(-0.02, -0.01)
Caribbean	282,262 (225,428–354,628)	760.72 (615.1-957.82)		343,061 (275,589–436,038)	747.58 (599.67-949.35)		-0.06*(-0.07, -0.04)
Central Asia	628,222 (513,737–781,019)	823.52 (672.31-1028.84)		769,645 (625,305–970,202)	799.56 (652.12-1005.85)		-0.09*(-0.11, -0.07)
Central Europe	1,082,188 (891,662–1,336,491)	945.6 (776.22-1173.19)		850,231 (694,792–1,047,733)	924.61 (758.75-1151.49)		-0.07*(-0.08, -0.07)
Central Latin America	1,556,715 (1,227,958–1,972,250)	871.6 (695.96 1111.08)		2,081,579 (1,643,105–2,677,105)	830.56 (658.49-1064.79)		-0.17*(-0.19, -0.15)
Central Sub-Saharan Africa	674,361 (548,945–829,229)	978.64 (804.6-1197.99)		1,409,106 (1,146,523–1,742,826)	920.37 (755.65-1123.02)		-0.21*(-0.23, -0.19)
East Asia	15,307,968 (8,879,963–24,470,261)	1213.26 (719.67-1901.9)		12,544,159 (6,977,460–20,355,095)	793.92 (464.24-1257.34)		-1.58*(-1.83, -1.33)
Eastern Europe	2,230,253 (1,819,107–2,778,925)	1051.84 (855.98-1318.65)		1,870,562 (1,516,334–2,341,536)	1042.06 (846.07-1310.53)		-0.02 (-0.05, 0.01)
Eastern Sub-Saharan Africa	2,249,586 (1,817,684–2,789,681)	977.92 (803.23-1205.51)		4,671,888 (3,720,299–5,818,830)	972.22 (794.01-1199.23)		-0.01(-0.07, 0.05)
High-income Asia Pacific	1,671,262 (1,369,598–2,062,540)	998.67 (824.11-1229.78)		1,516,461 (1,250,456–1,849,830)	975.91 (802.8-1206.1)		-0.08*(-0.09, -0.06)
High-income North America	2,474,652 (1,858,094–3,314,247)	899.01 (679.39-1187.11)		2,993,198 (2,292,968–3,904,041)	888.21 (678.97-1158.21)		-0.1 (-0.31, 0.12)
North Africa and Middle East	2,878,598 (2,284,318–3,683,835)	780.65 (629.51-989.52)		4,876,764 (3,860,897–6,279,738)	767.65 (617.55-976.53)		-0.05*(-0.06, -0.05)
Oceania	26,793 (21,067–34,570)	404.27 (318.19-522.75)		54,786 (43,138–70,623)	399.7 (313.87–516.3)		-0.04*(-0.05, -0.03)
South Asia	11,053,565 (8,762,605–14,053,013)	948.89 (759.38-1210.73)		17,320,008 (13,707,128–22,334,644)	929.75 (741.6-1189.58)		-0.06*(-0.09, -0.04)
Southeast Asia	2,594,546 (2,084,392–3,275,994)	531.78 (429.67-662.94)		3,586,439 (2,860,586–4,558,489)	524.21 (422.21-660.22)		-0.05*(-0.06, -0.03)
Southern Latin America	372,959 (305,062–459,347)	737.88 (607.45-908.14)		431,319 (354,085–529,090)	694.97 (567.41-858.64)		-0.21*(-0.22, -0.19)
Southern Sub-Saharan Africa	661,500 (532,176–821,916)	1130.9 (928.01-1391.91)		922,946 (749,220–1,146,255)	1129.01 (920.82-1395.58)		0.02 (-0.03, 0.07)
Tropical Latin America	1,413,991 (1,098,484–1,835,361)	884.73 (690.94-1139.66)		1,795,045 (1,348,735–2,375,058)	789.35 (601.24-1035.31)		-0.39*(-0.42, -0.37)
Western Europe	2,352,557 (1,950,215–2,868,554)	682.19 (563.21-839.81)		2,391,795 (1,975,557–2,917,212)	674.84 (555.74-832.37)		-0.03 (-0.06, 0.01)
Western Sub-Saharan Africa	2,263,625 (1,834,454–2,798,468)	1008.33 (831.77-1234.55)		5,184,810 (4,178,440–6,459,436)	990.05 (816.41-1215.93)		-0.06*(-0.08, -0.04)

ASIR: age-standardized incidence rate; AAPC: average annual percentage change. The AAPCs with asterisks (*) are statistically significant (*P* < 0.05)

**Table 2:** The death numbers and ASDR of foreign body in 1990 and 2019 with AAPCs over the 30 years

	1990			2019			
	Death number(95%UI)	ASDR (95%UI)		Death number(95%UI)	ASDR (95%UI)		AAPC, %(95%CI)
Global	124,633 (115,712–135,620)	2.39 (2.24–2.57)		114,413 (104,996–123,667)	1.55 (1.41–1.67)		-1.47*(-1.58, -1.37)
**Gender**							
Male	71,350 (64,438–78,579)	2.85 (2.63–3.07)		68,328 (62,934–74,254)	1.96 (1.8–2.12)		-1.26*(-1.38, -1.13)
Female	53,283 (48,484–58,966)	1.98 (1.82–2.16)		46,086 (41,753–50,010)	1.17 (1.07–1.28)		-1.78*(-1.83, -1.73)
**SDI rank**							
High SDI	17,508 (16,743–17,984)	2 (1.91–2.05)		29,149 (25,546–31,259)	1.71 (1.55–1.8)		-0.55*(-0.69, -0.41)
High-middle SDI	26,498 (25,000–28,255)	2.51 (2.37–2.69)		24,919 (22,842–26,633)	1.62 (1.49–1.75)		-1.44*(-1.78, -1.1)
Middle-SDI	46,100 (41,421–51,449)	2.66 (2.42–2.93)		27,745 (24,949–30,978)	1.35 (1.21–1.51)		-2.3*(-2.43, -2.17)
Low-middle SDI	23,586 (20,994–27,120)	1.9 (1.73–2.11)		18,498 (16,515–20,832)	1.26 (1.13–1.41)		-1.42*(-1.55, -1.3)
Low SDI	10,856 (9347–12,877)	2.12 (1.89–2.39)		14,024 (12,039–16,541)	1.59 (1.42–1.82)		-0.97*(-1.02, -0.92)
**GBD regions**							
Andean Latin America	7915 (6600–9303)	17.39 (14.83-20)		3192 (2455–4147)	5.21 (4.02–6.79)		-4.06*(-4.49, -3.63)
Australasia	179 (171–187)	0.91 (0.86–0.95)		294 (262–321)	0.75 (0.68–0.82)		-0.62*(-1.13, -0.11)
Caribbean	1701 (1407–2037)	4.77 (4.05–5.55)		1375 (1096–1674)	3.01 (2.35–3.71)		-1.58*(-1.95, -1.2)
Central Asia	1993 (1798–2196)	2.6 (2.38–2.82)		1819 (1596–2074)	2.05 (1.82–2.33)		-0.84*(-1.16, -0.52)
Central Europe	3022 (2927–3137)	2.66 (2.56–2.79)		2172 (1904–2454)	1.49 (1.29–1.69)		-1.99*(-2.27, -1.7)
Central Latin America	6892 (6429–7331)	4.09 (3.86–4.3)		5994 (5034–7189)	2.54 (2.13–3.05)		-1.63*(-1.78, -1.47)
Central Sub-Saharan Africa	1118 (896–1375)	2.53 (2.14–2.93)		1646 (1251–2095)	2.08 (1.65–2.56)		-0.68*(-0.79, -0.57)
East Asia	36,864 (32,049–42,648)	3.21 (2.81–3.69)		15,715 (13,731–17,660)	1.34 (1.17–1.53)		-2.96*(-3.2, -2.73)
Eastern Europe	7334 (6944–7736)	3.19 (3.02–3.37)		8808 (7766–9896)	3.47 (3.08–3.86)		0.42 (-0.8, 1.65)
Eastern Sub-Saharan Africa	3936 (3382–4627)	2.69 (2.37–3.03)		5239 (4516–6078)	2.07 (1.86–2.3)		-0.89*(-1.05, -0.74)
High-income Asia Pacific	4808 (4506–4991)	3.05 (2.83–3.22)		10,315 (8507–11,340)	2.12 (1.85–2.29)		-1.27*(-1.49, -1.05)
High-income North America	4776 (4553–4904)	1.54 (1.48–1.59)		9031 (8274–9459)	1.84 (1.74–1.93)		0.66*(0.34,0.97)
North Africa and Middle East	6016 (4904–7789)	1.57 (1.34–1.91)		4882 (4116–5784)	0.92 (0.78–1.08)		-1.83*(-1.9, -1.75)
Oceania	48 (40–77)	0.95 (0.81–1.31)		74 (59–128)	0.77 (0.63–1.11)		-0.73*(-0.87, -0.59)
South Asia	11,520 (9942–13,284)	1.15 (1.03–1.28)		11,787 (10,563–13,347)	0.86 (0.77–0.97)		-1.03*(-1.45, -0.61)
Southeast Asia	7746 (6583–9552)	1.9 (1.67–2.21)		7393 (6451–8488)	1.44 (1.26–1.65)		-0.93*(-1.09, -0.76)
Southern Latin America	3340 (3153–3537)	6.88 (6.5–7.28)		1427 (1323–1550)	2.07 (1.9–2.3)		-4.02*(-4.26, -3.78)
Southern Sub-Saharan Africa	1291 (1125–1444)	2.63 (2.35–2.89)		1358 (1182–1543)	1.91 (1.68–2.14)		-1.13*(-1.54, -0.73)
Tropical Latin America	2778 (2528–3175)	1.96 (1.81–2.18)		4312 (3924–4700)	2.16 (1.93–2.39)		0.35*(0.08,0.62)
Western Europe	7713 (7303–7997)	1.75 (1.67–1.82)		11,638 (10,166–12,498)	1.29 (1.17–1.36)		-1.07*(-1.19, -0.95)
Western Sub-Saharan Africa	3644 (2991–4422)	2 (1.71–2.31)		5940 (4758–7356)	1.71 (1.4-,2.03)		-0.54*(-0.63, -0.46)

ASDR: age-standardized death rate; AAPC: average annual percentage change. The AAPCs with asterisks (*) are statistically significant (P < 0.05)

### Trends by SDI

Regarding different SDI regions, the incidence number of FBs was the highest in the middle SDI regions, with 20,374,554 (95%UI 14,814,836–27,777,166) in 2019. The incidence number increased in high, high-middle, middle, low-middle, and low SDI regions, compared with 1990. In 2019, the highest ASIR was reported in the high SDI regions. Of note, the ASIR showed an uptrend in recent years in five SDI regions. especially in high, high-middle, and middle SDI regions. The upward trend of the ASIR in the high SDI region was divided into two stages, 2010–2017 (APC = 1.56%, *P* < 0.05) and 2017–2019 (APC = 3.69, *P* < 0.05); In the high-middle SDI region, the ASIR also increased from 2010, which was divided into two stages, 2010–2015 (APC = 1.29%, *P* < 0.05) and 2015–2019(APC = 2.22, *P* < 0.05); In middle SDI region, the ASIR showed an upward trend from 2011 to 2019 (APC = 2.36, *P* < 0.05). In 2019, the number of deaths and ASDR were highest in the high SDI regions. In all SDI regions, the ASDR showed a downward trend with significant decreases in AAPCs from 1990 to 2019 (Tables 1 and 2; Fig. 1, Supplementary material 1).

### Regional trends

**Fig. 2:**
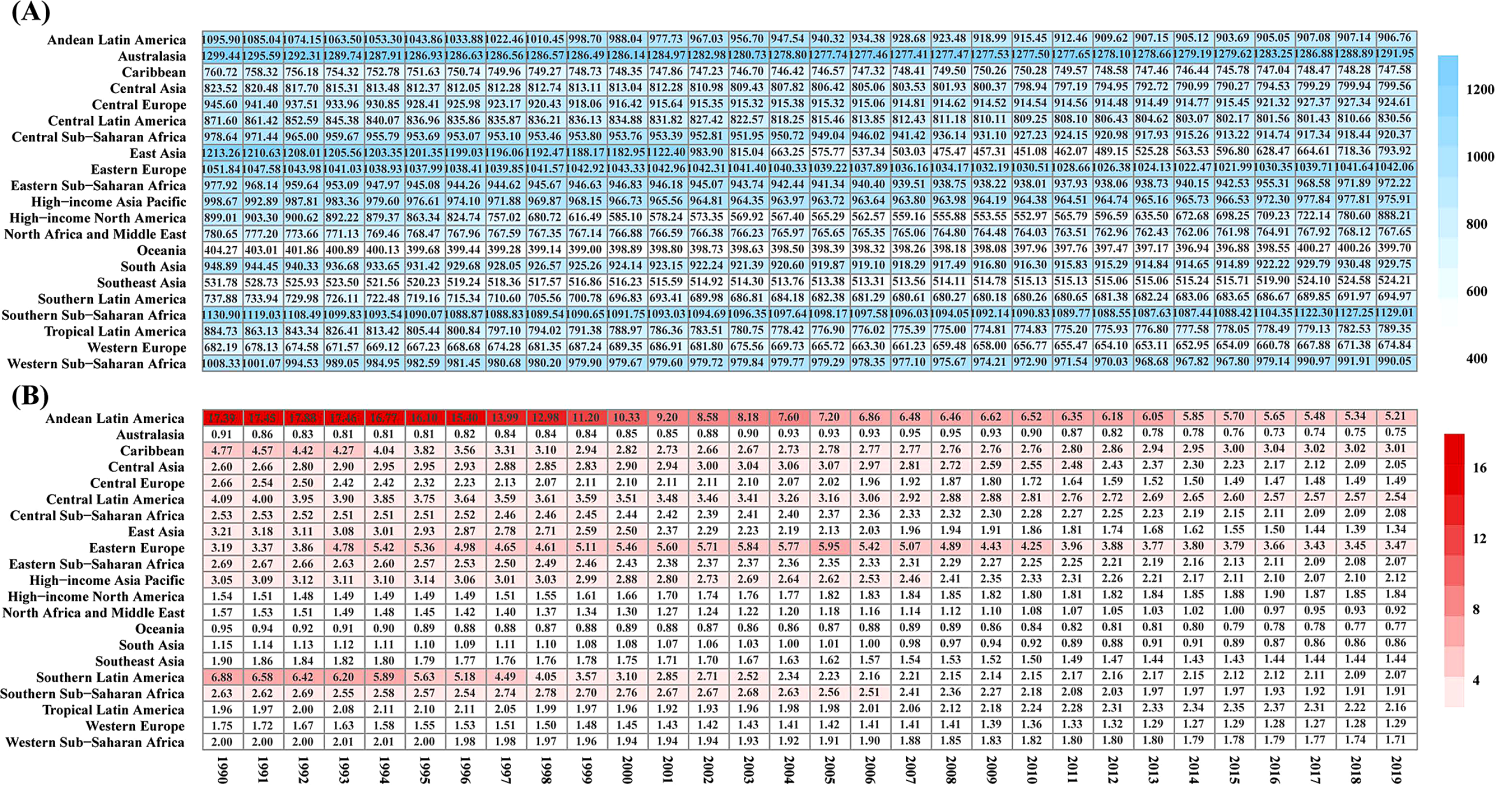
The ASIR and ASDR of foreign bodies in 21 regions from 1990 to 2019. (**A**) ASIR, (**B**) ASDR. ASIR: age-standardized incidence rate; ASDR: age-standardized death rate

At the regional level, the incidence number of FBs showed an upward trend from 1990 to 2019, except for Central Europe, High-Income Asia Pacific, Eastern Europe, and East Asia. The largest increase was in Western Sub-Saharan Africa from 2,263,625 (95% UI 1,834,454–2,798,468) in 1990 to 5,184,810 (95% UI 4,178,440–6,459,436) in 2019, and the highest incidence number was in South Asia with 17,320,008 (95% UI 13,707,128–22,334,644) and East Asia with 12,544,159 (95% UI 6,977,460–20,355,095). The ASIR in other regions decreased from 1990 to 2019, except for Southern Sub-Saharan Africa (AAPC = 0.02%, *P* > 0.05), and the highest decline was in East Asia (AAPC=-1.58%, *P* < 0.05). The highest ASIR in 2019 was in Australasia with 1291.95 (95%UI 1050.1-1586.41) per 100,000 population and Southern Sub-Saharan Africa with 1129.01 (95%UI 920.82-1395.58) per 100,000 population (Table 1; Fig. 2). The largest increase in deaths from FBs between 1990 and 2019 was in the High-income Asia Pacific from 4808 in 1990 to 10,315 in 2019, while the highest number of deaths was in East Asia, with 15,715 (95%UI 13,731–17,660) in 2019. The ASDR showed a downward trend in other regions, except in Eastern Europe (AAPC = 0.42%, *P* > 0.05), High-income North America (AAPC = 0.66%, *P* < 0.05), and Tropical Latin America (AAPC = 0.35%, *P* < 0.05). The highest ASDR in 2019 was in Andean Latin America with 5.21 (95%UI 4.02–6.79) per 100,000 population and in Southern Latin America with 2.07 (95%UI 1.90–2.30) per 100,000 population (Table 2; Fig. 2).

### National and territorial trends

**Fig. 3:**
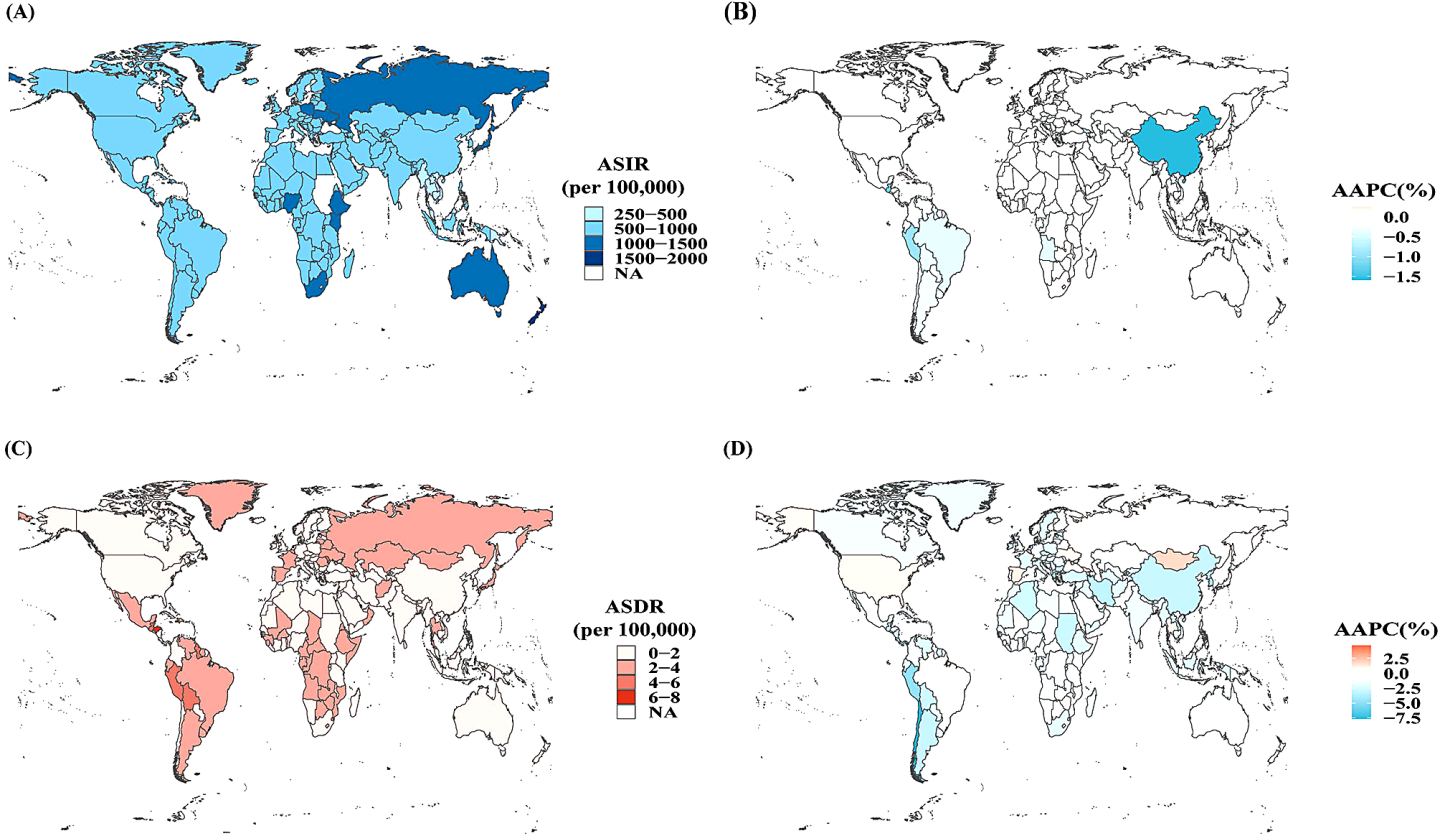
Global burden and trends of foreign bodies for 204 countries and territories. (**A**) ASIR in 2019, (**B**) AAPC of ASIR from 1990 to 2019, (**C**) ASDR in 2019, (**D**) AAPC of ASDR from 1990 to 2019. ASIR: age-standardized incidence rate; ASDR: age-standardized death rate. AAPC: average annual percentage change

At the national and territorial levels, New Zealand showed the highest ASIR of FBs, followed by South Africa, and Australia in 2019. The lowest ASIR was in the Democratic People’s Republic of Korea, followed by Taiwan (Province of China) and Kiribati in 2019. The joinpoint regression indicated that only 48 of the 204 countries reported a significant increase in the ASIR, with Cuba (AAPC = 0.26%, *P* < 0.05) reporting the largest increase, followed by Brunei Darussalam (AAPC = 0.24%, *P* < 0.05), and the Maldives (AAPC = 0.23%, *P* < 0.05) from 1990 to 2019. The largest decline in the ASIR occurred in China (AAPC = -1.60%, *P* < 0.05), followed by Guatemala (AAPC = -1.21%, *P* < 0.05), and Peru (AAPC = -0.91%, *P* < 0.05) in the same period. The highest ASDR was in Honduras, followed by Bolivia (Plurinational State of Bolivia) and Peru in 2019. From 1990 to 2019, the largest increase in the ASDR was in Cabo Verde (AAPC = 4.68%, *P* < 0.05), followed by Belgium (AAPC = 2.30%, *P* < 0.05) and Switzerland (AAPC = 2.08%, *P* < 0.05) in 2019. The largest decrease in the ASDR was in Chile (AAPC = -7.74%, *P* < 0.05) between 1990 and 2019 (Fig. 3). In addition, we conducted correlation analysis at the national or territorial level and found that the ASIR (*R* = -0.16, *P* < 0.05) and ASDR (*R* = -0.16, *P* < 0.05) for FBs in 2019 were significantly negatively correlated with the SDI index. (Supplementary material 2). This shows that the burden in low SDI regions was high and may indicate that health stakeholders and policy-makers in these areas need to make greater efforts in policies and medical intervention and prevention.

### Trends by sex and age

**Fig. 4:**
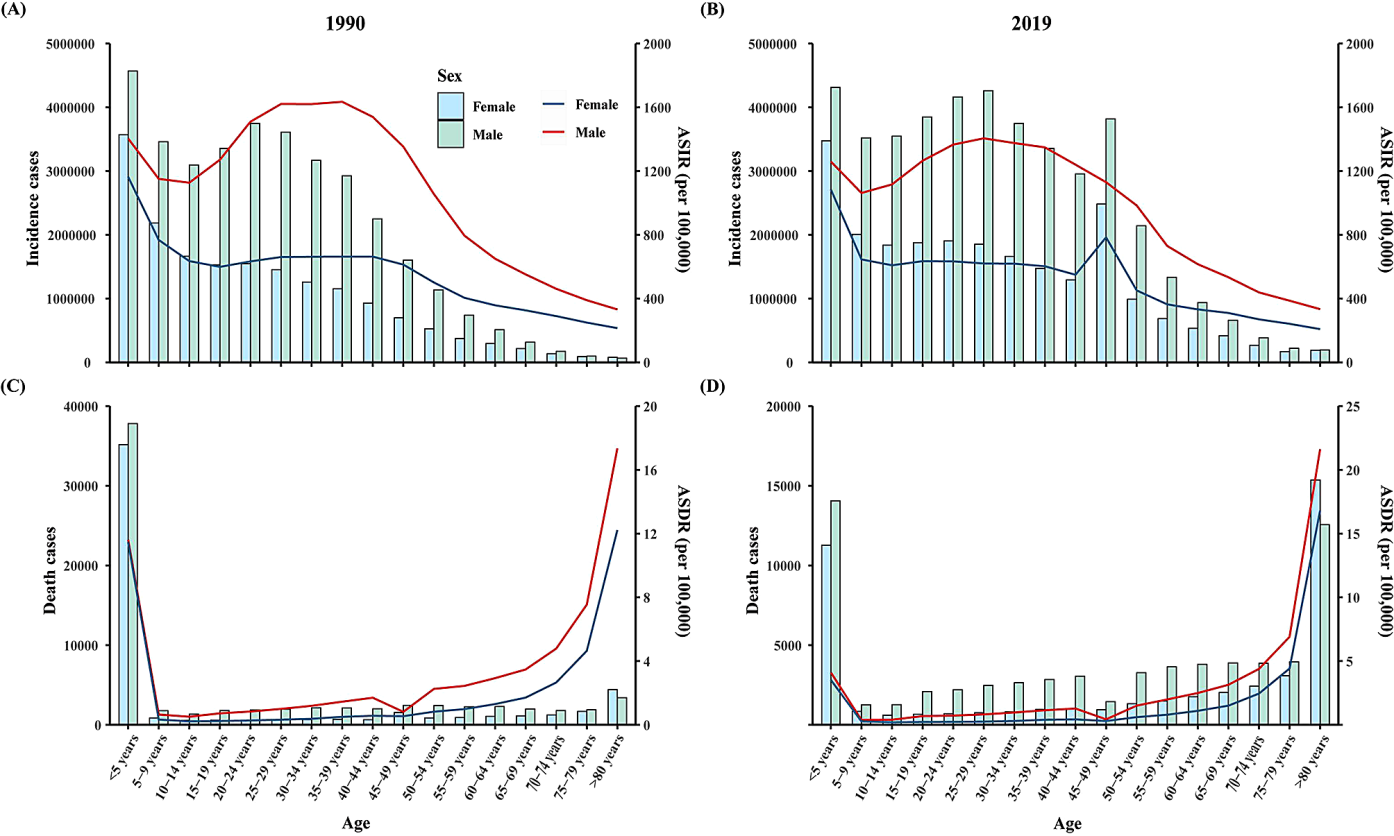
Global incidence and death cases and rate due to foreign bodies in 1990 and 2019, by age group. (**A**) incidence cases and rate in 1990, (**B**) incidence cases and rate in 2019, (**C**) death cases and rate in 1990, (**D**) death cases and rate in 2019

Based on sex groups, the incidence number of FBs for males and females in 2019 was 43,408,573 (95%UI 32,858,330–57,276,213) and 23,113,947 (95%UI 18,531,029–29,212,350), respectively. The ASIR for males was slightly higher than that for females, and the ASIRs for both males (AAPC = − 0.41%, *P* < 0.05) and females (AAPC = − 0.14%, *P* < 0.05) showed a downward trend from 1990 to 2019. In 2019, the number of deaths from FBs was 71,350 (95%UI 64,438–78,579) among males and 53,283 (95%UI 48,484 − 5896) among females. The trends of ASDRs among males and females were similar to those of ASIRs in the same period. (Tables 1 and 2; Figs. 1 and 4).

Worldwide and in different SDI regions, age was divided into five age groups: < 5 years old, 5–14 years old, 15–49 years old, 50–69 years old, and 70 + years old. There has been an overall downward trend across all age groups globally from 1990 to 2019. Of note, the global incidence rate of FBs showed an uptrend in recent years in all age groups. (Supplementary material 3, Supplementary material 4, Supplementary material 5). In terms of the death rate, notably, between 1990 and 2019, the death rate of the under-5 age group (AAPC= -3.76%, *P* < 0.05) decreased globally. The over-70 age group showed an upward trend worldwide (AAPC = 0.72%, *P* < 0.05), especially in high (AAPC = 1.03%, *P* < 0.05) and high-middle SDI regions (AAPC = 1.87%, *P* < 0.05) (Supplementary Material 6, Supplementary Material 7). Based on the trends, we further divided all age groups (0–4 years, 5–9 years … over 80 years) in the world. As shown in Fig. 4, in 2019, of note, the number and rate of incidence for males in the 5–44 age group were approximately twice that for females. In this age group, the number and rate of incidence rate among females were relatively stable, while the number and rate of incidence among males first increased and then decreased, reaching a peak in the 25–29 age group. In addition, the number and rate of incidence in the 0–4 age group was higher for both sexes. Among females, the incidence was higher in the 45–49 age group. The number of deaths and death rates for both sexes in 1990 and 2019 had a U-shaped association with all age groups. Notably, in 2019, the highest number of deaths for both males and females was in the under-5 and over 80 age groups. The highest death rates for both sexes were recorded in the under-5 and over-70 age groups. (Fig. 4)

### Trends by different diseases

**Fig. 5:**
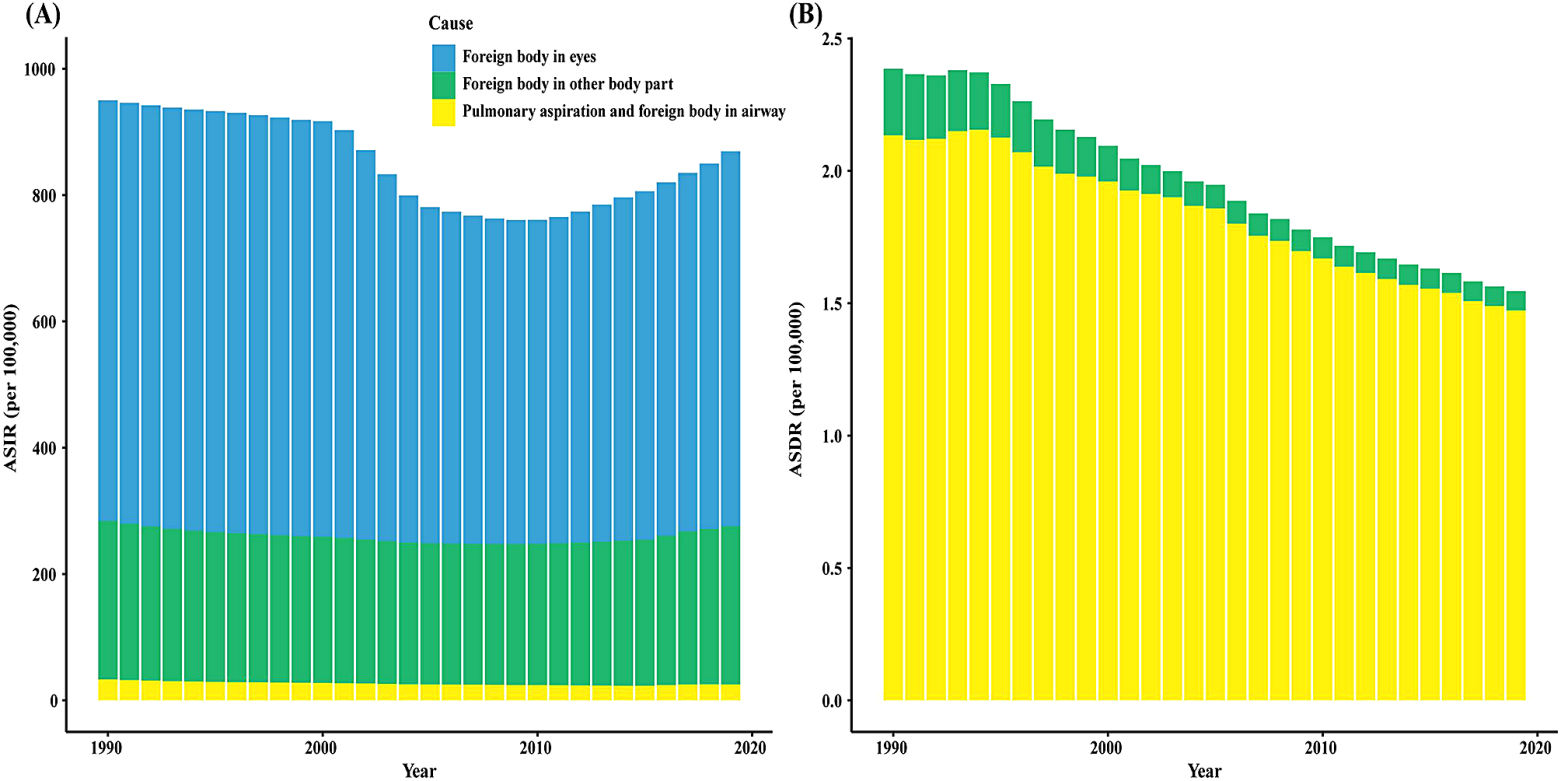
Global trends in ASIR and ASDR for different types of foreign bodies, 1990–2019. (**A**) ASIR, (**B**) ASDR. ASIR: age-standardized incidence rate; ASDR: age-standardized death rate

FBs include FBs in the eyes, FBs in other body parts, and PAFBA. Among them, the ASIR of FBs in eyes was the highest, and it showed an uptrend in recent years, reaching 593.26 (95%UI 416.01-812.51) per 100,000 population in 2019. No deaths from ocular FBs were reported. Notably, the ASIR of PAFBA was the lowest, with 25.13 (95%UI: 18.78–35.37) per 100,000 population, but the ASDR was the highest, with 1.54 (95%UI 1.43–1.64) per 100,000 population in 2019. The ASDR of FBs in other body parts (AAPC = − 4.19%, *P* < 0.05) and PAFBA (AAPC = − 1.25%, *P* < 0.05) showed a downward trend from 1990 to 2019 (Fig. 5, Supplementary material 8, Supplementary material 9).

### Spatial-temporal clustering

**Fig. 6:**
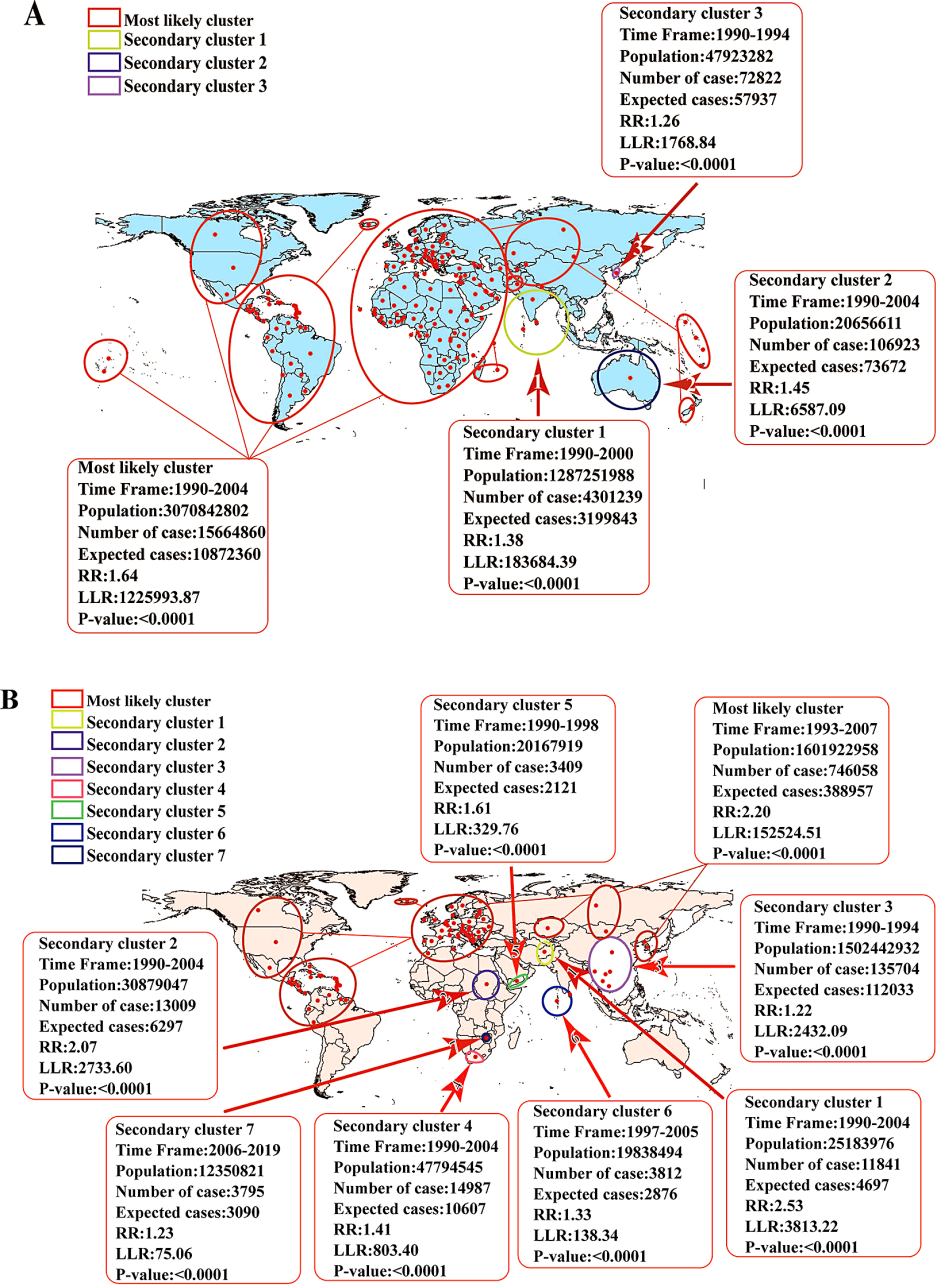
The spatial and temporal aggregation trend of pulmonary aspiration and foreign bodies in airway cases reported worldwide from 1990 to 2019. (**A**) Spatial and temporal of incidence cases (**B**) Spatial and temporal of death cases. RR: relative risk LLR: the log-likelihood ratio

As shown in the analysis, the ASIR of PAFBA was low, but the ASDR was the highest. Therefore, we further analyzed the temporal and spatial aggregation of PAFBA, providing a theoretical basis for further prevention and treatment. Geographic variation in the time trend analysis from 1990 to 2019 showed the incidence of four significant PAFBA clusters, including one most likely cluster and three secondary clusters. Between 1990/1/1 and 2004/12/31, the strongest aggregation regions were in Trinidad and Tobago, Grenada, and other countries (RR = 1.64, LLR = 1225993.87, *P* < 0.05). The number of incidences due to PAFBA reported in this region was 1,564,860, and the number of expected cases was 10,872,360。 Based on the spatial-temporal aggregation analysis of PAFBA deaths, there was one most likely cluster and seven secondary clusters. The strongest aggregation region had a total radius of 8181.64 km and included 82 countries, such as Canada, Cuba, and Mexico (RR = 2.20, LLR = 152524.51, *P* < 0.05). The number of PAFBA deaths reported in this region was 746,058 and the number of expected cases was 388,957, between 1993/1/1 and 2007/12/31. (Fig. 6). Through cluster analysis, we clarified the temporal and spatial distribution of the most likely cluster of PAFBA, further analyzed the reasons for these differences according to the obtained results, and proposed targeted measures to prevent and treat the disease.

## Discussion

In this research report, we used data from the GBD 2019 database to describe the global burden and time trend of FBs from 1990 to 2019. From 1990 to 2019, the global ASIR and ASDR of FBs decreased, but after 2010, the ASIR showed an upward trend. In terms of sex, the ASIR and ASDR of males were higher than those of females. The global incidence rate of FBs was higher among children, adolescents, and young adults, while the number of deaths was higher among children and elderly individuals. Notably, the global incidence rate of FBs in the 0–49 years age group showed an upward trend in recent years, while the death rate among people 70 and older showed an upward trend from 1990 to 2019. Among the different types of FBs, the ASIR of FBs in eyes was the highest, and the ASDR of PAFBA was the highest. We further analyzed the temporal and spatial aggregation of PAFBA and found that the strongest aggregation regions of incidence were in Trinidad and Tobago, Grenada, and other countries, and the strongest aggregation regions of deaths were in 82 countries, such as Canada, the United States, and Mexico.

We found that the global ASIR of FBs has increased since 2010, especially in high, high-middle, and middle SDI regions, which may be closely related to socioeconomic development, changes in people’s lifestyles and diets, and the development and progress of medical diagnostic technology. A study in Korea found that the incidence of FBs is increasing yearly, which is consistent with our research results [25]. China, with a population of 1.42 billion, is the most populous country in the world. Compared with other countries, the ASIR in Taiwan Province, China, was relatively low, and the ASIR in the Chinese mainland was very high, but the decline rate was the largest. In recent years, China’s economy has expanded rapidly, medical conditions have improved, traditional handicrafts have been gradually automated, laws and regulations have been implemented, people’s awareness of self-protection has been enhanced, and the working environments of workers have been greatly improved [26–29]. Although our country has a large population, the population is aging, and the incidence of FBs remains high.

In the GBD 2019, the ASIR and ASDR among men were higher than those among women. This may be explained by the fact that men may be more active, and they are more likely to be exposed to various dangerous products and situations [2]. They are curious, prefer to explore unknown areas, and are more likely to be interested in dangerous goods. Males may prefer to eat hard food, such as meat and nuts, which can easily cause FBs to become stuck in the throat or esophagus, thus increasing the risk of FBs. Finally, men are generally engaged in more dangerous jobs, such as construction, farming, and mechanics. There are many dangerous products and situations in these working environments, which are likely to lead to FB-related accidents. To reduce the risk of FBs among males, all levels of society should strengthen publicity and safety education, pay attention to eating habits, avoid eating food that is too hard or too large, strengthen personal protection during work, wear protective equipment, and avoid preventable FBs [2, 30]. For example, in 2009, China promulgated the Interim Provisions on Supervision and Supervision of Occupational Health in the Workplace to ensure legal prevention, control, and elimination of hazards to reduce the occurrence of FB incidents at the root and make the diagnosis and treatment of FBs more standardized [29]. At the same time, family safety should be improved. Other countries may also formulate laws and regulations in line with national conditions according to their actual social, economic, and medical development.

The global incidence rate of FBs was higher among children, adolescents, and young adults. However, the death rate was higher among children under 5 years old and people 70 and older. Because of children’s different cognitive abilities and anatomical structure, curiosity about new things, rapid intake of food, and the sale of dangerous toys, children are more likely to ingest FBs [8, 31]. In addition, some studies have found that bronchial FB inhalation is most likely to occur in infants under 5 years old and elderly individuals, and this kind of FB ingestion has a high death rate [1, 32]. Our study also found that the death rate of people 70 and older showed an uptrend, which may be attributed to the aging population. Therefore, we need to pay more attention to children and elderly people and take targeted measures for the prevention and control of emergency FB incidents in the future. First of all, we can strengthen the cultivation of children’s safety awareness through preschool education and advocate eye examinations for elderly individuals. Second, it is necessary to strengthen family supervision. We can strengthen the safety of children and elderly people through safety education for their guardians. Third, at the social level, we can also set up a home for the protection of children and elderly individuals, strengthen the public’s understanding of the dangers of FBs, and popularize preventive measures through safety information. Finally, medical institutions also need to improve the level of diagnosis and treatment of FBs and diagnose FB cases in time to avoid aggravation [8, 28].

Among the different kinds of FBs, the ASIR of FBs in eyes was the highest. With the aging of the population, the delay in retirement age, cataracts, age-related macular degeneration, and other related eye diseases lead to poor vision, which makes it impossible to avoid FBs in the eyes during work. Therefore, it is necessary to protect the eyes, strengthen eye hygiene, and have regular eye examinations for the prevention of ophthalmic diseases. Some studies have also found that setting off fireworks and firecrackers can easily cause eye injuries [2, 33]. It is necessary not only to protect one’s safety but also to strengthen the awareness of eye protection for people involved in dangerous work, such as fireworks. Public health personnel should prevent elderly people from engaging in high-risk jobs and provide economic security; it is also suggested to use personal protective glasses when setting off fireworks and firecrackers to reduce the harm of fireworks and firecrackers to the eyes [34].

In addition, the ASDR of PAFBA was the highest. Some studies have found that PAFBA is the most likely to cause complications, and some complete FBs in the airway easily cause suffocation [35]. If treatment is not timely, people may die in a few minutes, so it is necessary to make an early diagnosis and take corresponding emergency measures to avoid complications. With the rapid development of the social and economic environment, emergency specialties and emergency systems are gradually improving, and the whole society is fully concerned. With the development of medical imaging technology, the success rate of bronchoscopy in clearing airway FBs is as high as 99.2%, which greatly shortens the operation time and reduces the incidence of various complications during and after the operation, such as hypoxemia, airway mucosal injury and bleeding after treatment, and laryngeal edema. The application of computed tomography(CT)before and after removal can be used to evaluate the presence, affected parts, and secondary changes (for example, pneumonia or atelectasis) of FBs in the airway, and to evaluate the removal of FBs [36–40]. However, the ASDR was still high, so we further analyzed the temporal and spatial aggregation of PAFBA. In our research, the areas with the highest incidence rates were North America, South America, Europe, and Africa, and the areas with the highest death rates were Europe, the junction of North America and South America. However, incidence and death aggregation occurred before 2004 and 2007, respectively, which may be due to public health personnel’s increased awareness of the disease, strengthened publicity and education, and the popularization of first-aid knowledge related to FBs in the trachea [5]. The CHOP community intervention experimental study designed in Italy aims to reform the school system to give lectures on the dangers of inhaling FBs for children, parents, guardians, and faculty, and hopes that the program will be implemented in other countries; The United States and Sweden formulate laws and regulations to prevent suffocation; Canada uses the Internet as a medium to transmit the knowledge of preventing FB suffocation; Colombia has established an open and free educational website to raise people’s awareness of the dangers of FB inhalation; to reduce the occurrence of FB events; China formulated relevant laws and regulations to reduce FB events through work [41, 42].

Our research results showed that since 2010, the ASIR of FBs has increased worldwide, and FBs have become a global public health problem that needs urgent attention. Among the different types of FBs, PAFBA is the most obvious, and prevention and timely treatment can reduce the mortality of PAFBA. The incidence and mortality of FBs vary greatly at different ages, so we should further evaluate and implement more powerful and comprehensive prevention and publicity measures for the most affected populations. It is worth mentioning that there are obvious differences in the incidence of FBs in different SDI regions. The significant differences between the ASIRs and ASDRs in different regions may be related to the rapid development of social and economic conditions, further improvement of basic health care preventive measures, occupational exposure hazards, and even regional differences in culture. Given the global nature of FB incidents, we think it may be beneficial to strengthen exchanges and cooperation among countries on the latest progress in the prevention, treatment, and research of FB incidents, further analyze the reasons for these differences, and draw lessons from countries and regions with low ASIRs to formulate corresponding policies and regulations to narrow the gap.

### Limitations

This study had several limitations. First, personal reasons (low education and fear of medical intervention) lead to underreporting of FB data globally. Some patients with FBs have mild or no symptoms, and they do not actively seek medical treatment. These patients with mild or no symptoms are not easy to diagnose when they seek medical treatment. Therefore, potential strategies to improve early detection and diagnosis, such as increasing public awareness or improving diagnostic tools, should be considered. Second, due to the limitation of medical technology, the early diagnosis rate of FBs may be low, especially in some developing countries. Although the data has been corrected by modeling, it may still be different from the actual data. This may be based on the research results, and the global medical and health strategies and interventions may not be fully applicable to the current situation in some developing countries. Third, during the COVID-19 pandemic, the use of telemedicine was promoted, which may have helped the diagnosis and treatment of FB incidents, and may also have promoted the coordination and cooperation of various departments at the national, regional, and global levels, thus having an impact on FB incidents. However, the FB data in this database is updated to 2019, lacking COVID-19 data, so it was impossible to accurately evaluate the influence of COVID-19 on FB trends. Finally, the research results obtained by secondary analysis of GBD data were influenced by the quality and quantity of data used in the modeling process. We lack additional detailed covariate data to control the deviation. In the future, the comprehensive evaluation of the disease burden needs to fully consider social, economic, family, and individual factors. To provide more accurate results, more relevant data need to be collected to address these limitations.

## Conclusion

This study showed that the ASIR of FBs gradually increased from 2010 to 2019, especially in high, high-middle, and middle SDI regions. The incidence rate of FBs was higher among children, adolescents, and young adults, while the number of deaths was higher among children and elderly individuals. This requires the government and healthcare planners to formulate practical and targeted policy responses according to the characteristics of morbidity and mortality in different regions and different age groups. These measures include developing global health care and strengthening the implementation of basic social medical care. Among the different types of FBs, the ASDR of PAFBA was the highest. It is necessary to strengthen the exchange, cooperation, and experience sharing of prevention of PAFBA among countries and reduce the ASDR. However, this study failed to further explore the influence of the COVID-19 pandemic on FB incidents, which is worthy of further research by researchers.

### Electronic supplementary material

Below is the link to the electronic supplementary material.

Supplementary Material 1

## Data Availability

Publicly available datasets were analyzed in this study. The data can be found here: http://ghdx.healthdata.org/gbd-results-tool.

## Abbreviations

AAPC: average annual percent change
ASIR: the age-standardized incidence rate
ASDR: the age-standardized death rate
CHOP: CHOking Prevention
CI: confidence interval
COVID-19: the Corona Virus Disease 2019
CT: computed tomography
FBA: Foreign body aspiration
FBs: Foreign bodies
FoBIs: Foreign body ingestions
GBD: Global Burden of Disease
GBD 2019: The Global Burden of Disease Study 2019
GHDx: The SDI of the Global Health Data Exchange
IHME: the Institute for Health Metrics and Evaluation
IOFBs: intraocular foreign bodies
LLR: the log-likelihood ratio
PAFBA: pulmonary aspiration and foreign body in the airway
SDI: sociodemographic index
UIs: uncertainty intervals
