# Supplementary material for: The global, regional, and national burden of foreign bodies from 1990 to 2019: a systematic analysis of the global burden of disease study 2019
Source: BMC Public Health. 2024 Jan 31;24:337. doi: 10.1186/s12889-024-17838-x (PMC10829478; doi:10.1186/s12889-024-17838-x)
Supplement: Supplementary file 1 — Supplementary Material 1 [file 12889_2024_17838_MOESM1_ESM.pdf]

Supplementary material 1. Long-term trend analysis of foreign body disease burden in different regions from 1990 to 2019

	ASIR		ASDR	
	year	APC (%, 95% CI)	year	APC (%, 95% CI)
Global				
Period 1	1990-2001	-0.39 (-0.40, -0.37)	1990-1994	0.0003 (-0.33, 0.33)
Period 2	2001-2004	-4.37*(-4.65, -4.08)	1994-1998	-2.40*(-2.91, -1.89)
Period 3	2004-2007	-1.32*(-1.62, -1.03)	1998-2005	-1.53*(-1.71, -1.36)
Period 4	2007-2010	-0.31*(-0.61, -0.01)	2005-2010	-2.08*(-2.40, -1.75)
Period 5	2010-2015	1.20*(1.11, 1.30)	2010-2019	-1.32*(-1.41, -1.22)
Period 6	2015-2019	1.91*(1.81, 2.00)		
High SDI				
Period 1	1990-1995	-0.52*(-0.68, -0.36)	1990-1996	-0.72*(-0.92, -0.53)
Period 2	1995-2000	-2.98*(-3.2, -2.76)	1996-1999	0.25 (-0.93, 1.45)
Period 3	2000-2010	-0.21*(-0.28, -0.15)	1999-2006	-0.36*(-0.56, -0.16)
Period 4	2010-2017	1.56*(1.44, 1.69)	2006-2014	-1.16*(-1.32, -1.00)
Period 5	2017-2019	3.69*(2.95, 4.44)	2014-2019	-0.10 (-0.36, 0.17)
High-middle SDI				
Period 1	1990-2001	-0.20*(-0.27, -0.13)	1990-1994	3.38*(2.34, 4.43)
Period 2	2001-2005	-5.85 (-6.31, -5.38)	1994-1998	-3.49*(-5.02, -1.93)
Period 3	2005-2010	-0.89*(-1.20, -0.58)	1998-2005	-0.39 (-0.92, 0.15)
Period 4	2010-2015	1.29*(0.98, 1.61)	2005-2009	-4.28*(-5.80, -2.74)
Period 5	2015-2019	2.22*(1.91, 2.55)		
Middle SDI				
Period 1	1990-2001	-0.42*(-0.48, -0.35)	1990-1993	-1.19*(-1.66, -0.71)
Period 2	2001-2004	-7.93*(-8.82, -7.03)	1993-1996	-2.72*(-3.65, -1.79)
Period 3	2004-2007	-3.03*(-3.97, -2.08)	1996-2001	-3.66*(-3.96, -3.37)
Period 4	2007-2011	-0.36*(-0.85, 0.12)	2001-2007	-3.01*(-3.22, -2.80)
Period 5	2011-2019	2.36*(2.25, 2.47)	2007-2012	-1.29*(-1.58, -0.99)
Period 6			2012-2019	-1.72*(-1.84, -1.59)
Low-middle SDI				
Period 1	1990-1993	-0.53*(-0.73, -0.34)	1990-1997	-1.79*(-1.92, -1.65)
Period 2	1993-2001	-0.19*(-0.25, -0.14)	1997-2002	-2.27*(-2.59, -1.95)
Period 3	2001-2004	-1.90*(-2.29, -1.51)	2002-2011	-1.11*(-1.22, -1.00)
Period 4	2004-2009	-0.33*(-0.45, -0.20)	2011-2014	-0.39 (-1.41, 0.64)
Period 5	2009-2014	0.32 (0.20, 0.45)	2014-2019	-1.23*(-1.45, -1.00)
	2014-2019	0.99*(0.9, 1.08)		
Low SDI				
Period 1	1990-1994	-0.62*(-0.77, -0.46)	1990-1994	-0.68*(-0.85, -0.50)
Period 2	1994-2014	-0.06*(-0.07, -0.04)	1994-2001	-1.12*(-1.21, -1.03)
Period 3	2014-2019	0.57*(0.45, 0.68)	2001-2006	-0.74*(-0.91, -0.56)
Period 4			2006-2011	-1.30*(-1.47, -1.12)
Period 5			2011-2019	-0.93*(-0.99, -0.87)

ASIR: age-standardized incidence rate; ASDR: age-standardized death rate; APC: annual percentage change. The APCs with asterisks (*) are statistically significant (P <0.05).

Supplementary material 2: The correlation between ASIR and ASDR and SDI in 204 countries

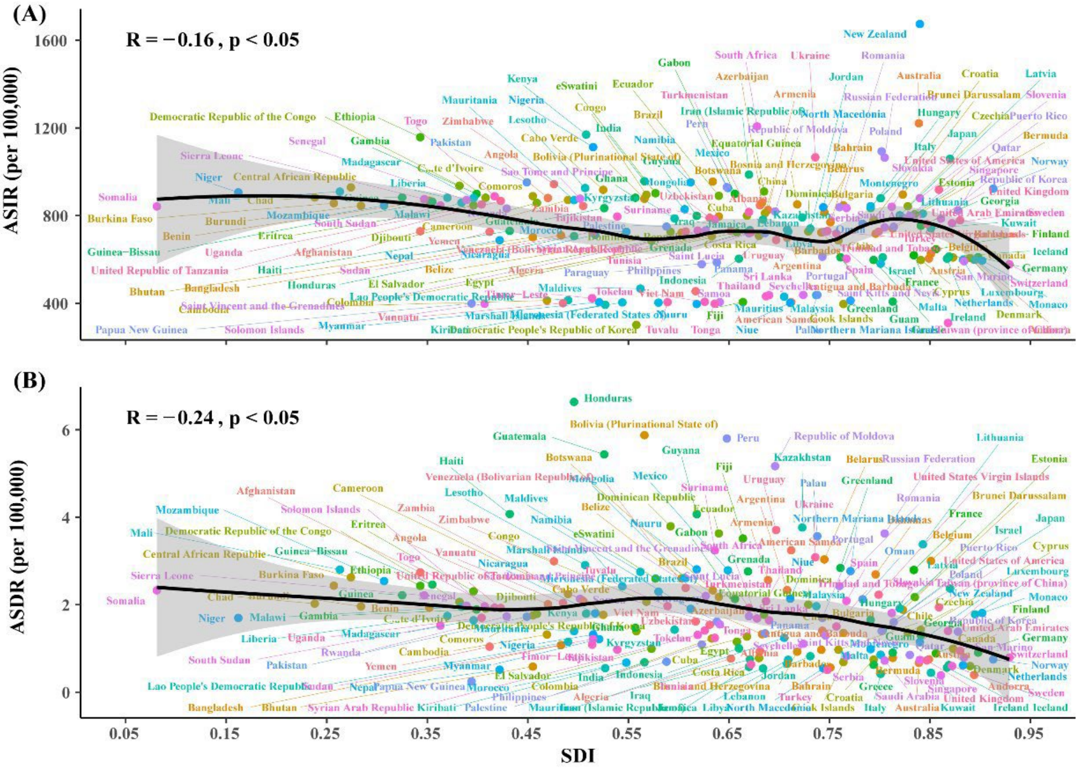

(A) ASIR, (B)ASDR. ASIR: age-standardized incidence rate; ASDR: age-standardized death rate; SDI: sociodemographic index.

Supplementary material 3. Long-term trend analysis of foreign body disease burden in different age groups from 1990 to 2019

	ASIR		ASDR	
	year	APC (%, 95% CI)	year	APC (%, 95% CI)
<5 years				
Period 1	1990-1994	-1.34*(-1.46, -1.22)	1990-1995	-3.19*(-3.48, -2.90)
Period 2	1994-2001	-0.70*(-0.76, -0.63)	1995-2001	-5.04*(-5.33, -4.76)
Period 3	2001-2004	-1.43*(-1.80, -1.06)	2001-2010	-3.36*(-3.50, -3.22)
Period 4	2004-2009	-0.52*(-0.64, -0.40)	2010-2016	-2.46*(-2.75, -2.17)
Period 5	2009-2015	0.31*(0.22, 0.39)	2016-2019	-5.89*(-6.52, -5.25)
Period 6	2015-2019	1.68*(1.56, 1.80)		
5-14 years				
Period 1	1990-1993	-0.92*(-1.06, -0.79)	1990-1999	-1.70*(-1.82, -1.59)
Period 2	1993-2001	-0.36*(-0.39, -0.32)	1999-2003	-3.63*(-4.22, -3.03)
Period 3	2001-2004	-2.16*(-2.43, -1.90)	2003-2007	-1.36*(-1.97, -0.74)
Period 4	2004-2009	-0.09*(-0.18,0.005)	2007-2010	-0.13 (-1.36, 1.12)
Period 5	2009-2015	0.69*(0.63, 0.75)	2010-2019	-1.95*(-2.06, -1.84)
Period 6	2015-2019	1.72*(1.64, 1.81)		
15-49 years				
Period 1	1990-1998	-0.18*(-0.25, -0.11)	1990-1994	4.10*(3.12, 5.08)
Period 2	1998-2001	-0.72*(-1.36, -0.07)	1994-1997	-2.13 (-5.01, 0.84)
Period 3	2001-2004	-5.35*(-5.97, -4.73)	1997-2004	-0.37 (-0.87, 0.13)
Period 4	2004-2007	-1.86*(-2.50, -1.22)	2004-2012	-2.58*(-2.97, -2.19)
Period 5	2007-2011	-0.08*(-0.40, 0.25)	2012-2019	0.11 (-0.29, 0.51)
Period 6	2011-2019	1.74*(1.67, 1.82)		
50-69 years				
Period 1	1990-2001	-0.16*(-0.20, -0.12)	1990-1994	3.67*(2.65, 4.71)
Period 2	2001-2005	-5.07*(-5.37, -4.77)	1994-1998	-2.27*(-3.80, -0.73)
Period 3	2005-2010	-1.10*(-1.29, -0.90)	1998-2003	0.83 (-0.17, 1.83)
Period 4	2010-2013	1.73*(1.09, 2.37)	2003-2012	-2.32*(-2.65, -1.98)
Period 5	2013-2019	2.50*(2.40, 2.61)	2012-2019	-1.12*(-1.54, -0.71)
70+ years				
Period 1	1990-1995	0.76*(0.61, 0.91)	1990-1999	1.28*(1.16, 1.39)
Period 2	1995-2001	-0.98*(-1.13, -0.83)	1999-2010	0.31*(0.21, 0.41)
Period 3	2001-2005	-3.33*(-3.66, -3.00)	2010-2019	0.68*(0.56, 0.80)
Period 4	2005-2010	-0.96*(-1.18, -0.75)		
Period 5	2010-2017	1.68*(1.56, 1.80)		
Period 6	2017-2019	2.63*(1.94, 3.33)		

SIR: age-standardized incidence rate; ASDR: age-standardized death rate; APC: annual percentage change. The APCs with asterisks (*) are statistically significant (P <0.05).

Supplementary material 4. Trends in the incidence rate of foreign bodies by age group in global and SDI regions, 1990-2019

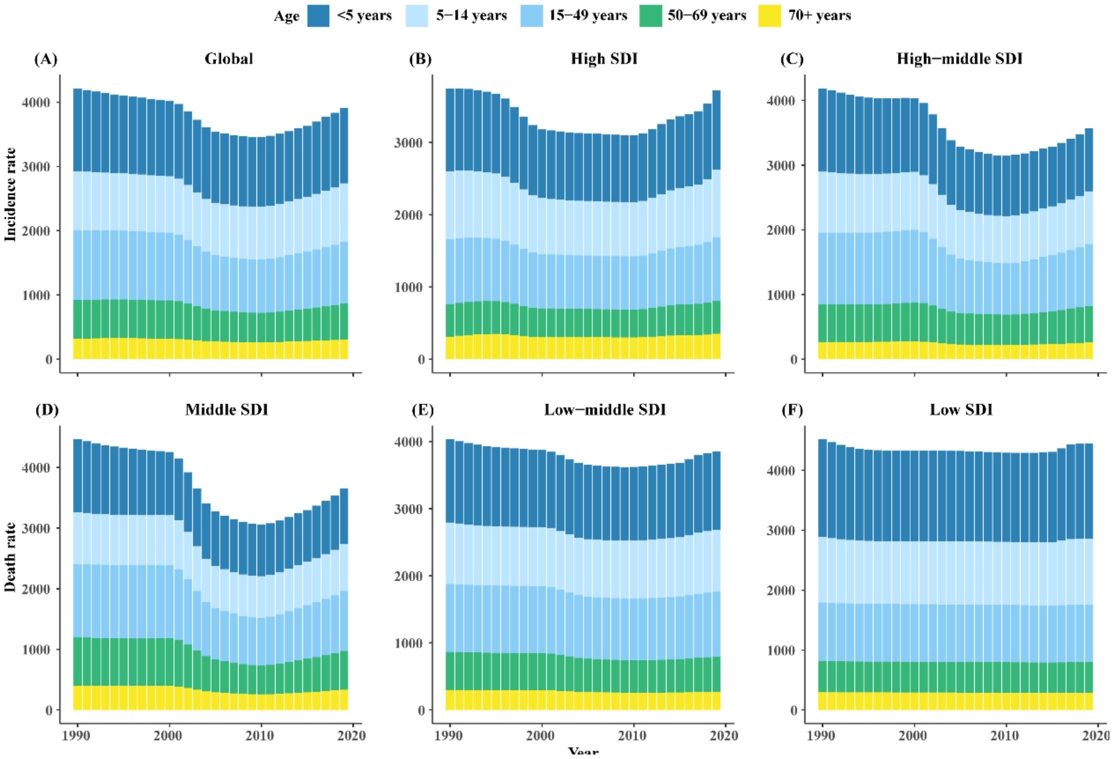

(A)in global, (B) high, (C) high-middle, (D) middle, € low-middle, (F)and low SDI regions. The populations were divided into five age groups: < 5 years, 5-14 years, 15- 49 years, 50-69 years, and 70+ years. SDI, sociodemographic index.

Supplementary material 5. The incidence rate of foreign bodies in 1990 and 2019 with AAPCs over the 30 years

	Incidence rate (95%UI)		AAPC, %(95%CI)
	1990	2019	
Global			
<5 years	1287.59 (965.22-1698.15)	1174.90 (870.01-1580.68)	-0.30*(-0.35, -0.25)
5-14 years	927.41 (612.37-1418.40)	912.17 (613.22-1343.67)	-0.06*(-0.09, -0.02)
15-49 years	1077.80 (676.70-1635.54)	960.10 (639.64-1401.19)	-0.42*(-0.54, -0.30)
50-69 years	602.65 (345.39-1027.72)	558.16 (320.75-943.85)	-0.28*(-0.36, -0.20)
70+ years	317.43 (207.49-513.56)	307.82 (206.27-486.04)	-0.13*(-0.21, -0.04)
High SDI			
<5 years	1142.81 (836.7-1582.13)	1097.20 (796.79-1555.44)	-0.17*(-0.25, -0.09)
5-14 years	940.39 (625.97-1386.39)	940.19 (617.64-1394.98)	-0.04 (-0.15, 0.06)
15-49 years	900.41 (625.45-1258.03)	875.64 (614.95-1202.32)	-0.12*(-0.19, -0.05)
50-69 years	449.81 (298.67-675.10)	457.15 (300.92-678.98)	0.01 (-0.12, 0.14)
70+ years	311.06 (226.97-434.94)	350.77 (262.07-471.27)	0.39*(0.03, 0.75)
High-middle SDI			
<5 years	1288.04 (957.25-1724.56)	973.11 (716.50-1325.48)	-0.97*(-1.02, -0.91)
5-14 years	942.01 (606.12-1470.67)	816.36 (545.82-1245.64)	-0.51*(-0.58, -0.43)
15-49 years	1108.75 (668.82-1721.14)	951.79 (588.38-1466.47)	-0.55*(-0.63, -0.47)
50-69 years	578.42 (321.31-1002.63)	566.77 (305.90-1010.39)	-0.09*(-0.17, 0.00)
70+ years	267.50 (167.71-451.03)	259.29 (163.03-440.27)	-0.11*(-0.18, -0.03)
Middle SDI			
<5 years	1202.45 (899.12-1584.41)	909.88 (659.75-1238.62)	-0.96*(-1.00, -0.93)
5-14 years	859.14 (540.88-1379.10)	781.12 (504.96-1217.08)	-0.34*(-0.46, -0.23)
15-49 years	1208.84 (691.60-1938.57)	990.22 (622.47-1509.22)	-0.71*(-0.83, -0.60)
50-69 years	792.37 (388.01-1510.02)	637.2 (326.14-1175.43)	-0.74*(-0.87, -0.62)
70+ years	403.76 (231.23-719.53)	333.5 (198.70-597.48)	-0.69*(-0.83, -0.56)
Low-middle SDI			
<5 years	1242.50 (935.52-1634.68)	1165.01 (860.66-1567.21)	-0.22*(-0.28,-0.16)
5-14 years	916.23 (625.82-1363.29)	922.68 (628.88-1364.88)	0.03 (-0.01,0.07)
15-49 years	1011.37 (680.95-1443.34)	969.90 (667.44-1361.67)	-0.14*(-0.2,-0.07)
50-69 years	567.33 (343.33-928.57)	520.20 (319.90-838.16)	-0.29*(-0.38,-0.2)
70+ years	297.39 (195.96-473.84)	272.49 (180.95-424.91)	-0.28*(-0.35,-0.22)
Low SDI			
<5 years	1633.20 (1231.60-2134.79)	1592.35 (1184.58-2116.51)	-0.07 (-0.17,0.02)
5-14 years	1096.84 (749.05-1578.14)	1102.06 (748.64-1585.06)	0.02 (-0.01,0.06)
15-49 years	973.44 (693.66-1309.97)	958.53 (684.65-1285.57)	-0.05*(-0.06,-0.04)
50-69 years	518.78 (342.57-788.54)	510.95 (336.18-778.54)	-0.05*(-0.06,-0.03)
70+ years	296.68 (210.43-433.18)	284.53 (201.40-417.73)	-0.14*(-0.15,-0.13)

AAPC: average annual percentage change. The AAPCs with asterisks (*) are statistically significant (P <0.05).

Supplementary material 6. Trends in the death rate of foreign bodies by age group in global and SDI regions, 1990-2019

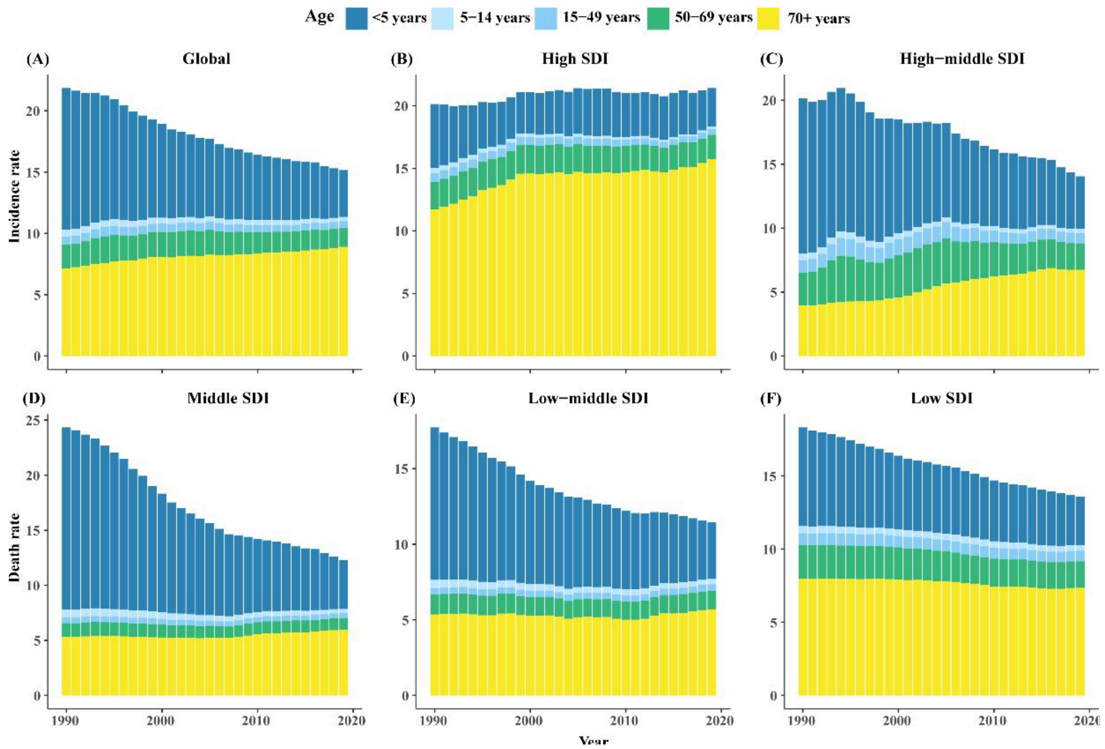

(A) in global, (B) high, (C) high-middle, (D) middle, (E) low-middle, (F)and low SDI regions. The populations were divided into five age groups: < 5 years,5-14 years, 15-49 years, 50-69 years, and 70+ years. SDI, sociodemographic index.

Supplementary material 7. The death rate of foreign bodies in 1990 and 2019 with AAPCs over the 30 years

	Death rate (95%UI)		AAPC, %(95%CI)
	1990	2019	
Global			
<5 years	11.55 (10.26-13.13)	3.82 (3.27-4.47)	-3.76*(-3.88, -3.64)
5-14 years	0.59 (0.55-0.65)	0.35 (0.31-0.39)	-1.84*(-2.01, -1.67)
15-49 years	0.65 (0.63-0.68)	0.56 (0.52-0.61)	-0.45*(-0.81, -0.10)
50-69 years	1.90 (1.84-1.97)	1.54 (1.44-1.64)	-0.68*(-0.99, -0.36)
70+ years	7.16 (6.58-7.52)	8.89 (7.65-9.57)	0.72*(0.66, 0.78)
High SDI			
<5 years	5.11 (4.82-5.43)	3.08 (2.76-3.41)	-1.76*(-1.94, -1.59)
5-14 years	0.41 (0.39-0.43)	0.19 (0.18-0.20)	-2.71*(-2.88, -2.53)
15-49 years	0.70 (0.67-0.71)	0.48 (0.46-0.51)	-1.26*(-1.34, -1.19)
50-69 years	2.20 (2.15-2.25)	1.93 (1.85-2.01)	-0.47*(-0.57, -0.37)
70+ years	11.72 (10.66-12.29)	15.73 (13.08-17.19)	1.03*(0.95, 1.12)
High-middle SDI			
<5 years	12.13 (10.77-13.82)	4.12 (3.48-4.82)	-3.65*(-4.04, -3.25)
5-14 years	0.54 (0.51-0.57)	0.31 (0.28-0.34)	-1.99*(-2.38, -1.59)
15-49 years	0.96 (0.93-0.99)	0.83 (0.76-0.90)	-0.41 (-1.32, 0.51)
50-69 years	2.55 (2.47-2.66)	2.06 (1.90-2.24)	-0.61 (-1.29, 0.07)
70+ years	3.95 (3.68-4.15)	6.74 (5.89-7.28)	1.87*(1.61, 2.14)
Middle SDI			
<5 years	16.52 (14.31-19)	4.43 (3.67-5.21)	-4.45*(-4.72, -4.17)
5-14 years	0.75 (0.69-0.81)	0.38 (0.33-0.43)	-2.36*(-2.54, -2.18)
15-49 years	0.52 (0.49-0.55)	0.47 (0.43-0.53)	-0.30*(-0.57, -0.03)
50-69 years	1.25 (1.18-1.33)	1.04 (0.93-1.15)	-0.62*(-0.78, -0.46)
70+ years	5.29 (4.83-5.66)	5.96 (5.23-6.53)	0.42*(0.29, 0.54)
Low-middle SDI			
<5 years	10.09 (8.66-11.95)	3.76 (3.11-4.53)	-3.34*(-3.48, -3.21)
5-14 years	0.54 (0.48-0.61)	0.35 (0.29-0.41)	-1.55*(-1.78, -1.33)
15-49 years	0.42 (0.39-0.46)	0.43 (0.38-0.48)	0.07 (-0.13, 0.26)
50-69 years	1.32 (1.22-1.43)	1.23 (1.10-1.36)	-0.25*(-0.37, -0.13)
70+ years	5.35 (4.82-5.89)	5.70 (5.08-6.20)	0.18*(0.06, 0.30)
Low SDI			
<5 years	6.74 (5.46-8.50)	3.31 (2.56-4.20)	-2.42*(-2.49, -2.36)
5-14 years	0.49 (0.39-0.60)	0.39 (0.32-0.48)	-0.76*(-1.02, -0.50)
15-49 years	0.79 (0.68-0.89)	0.71 (0.60-0.83)	-0.34*(-0.43, -0.24)
50-69 years	2.32 (2.05-2.60)	1.81 (1.58-2.08)	-0.85*(-0.94, -0.77)
70+ years	7.96 (7.00-8.93)	7.35 (6.60-8.08)	-0.28*(-0.39, -0.16)

AAPC: average annual percentage change. The AAPCs with asterisks (*) are statistically significant ($P < 0.05$).

Supplementary material 8. The incidence numbers and ASIR of different foreign bodies global in 1990 and 2019 with AAPCs over the 30 years

	1990		2019		
	Incidence number(95%UI)	ASIR(95%UI)	Incidence number(95%UI)	ASIR(95%UI)	AAPC, %(95%CI)
Foreign body in eyes	35789179 (23616153-50893363)	665.92 (442.23-944.10)	46628656 (32450161-64454053)	593.26 (416.01-325.52)	-0.43*(-0.53, -0.33)
Pulmonary aspiration and foreign body in the airway	1975532 (1482530-2772315)	33.01 (25.10-322.93)	1775385 (1339556-2468552)	25.13 (18.78-35.37)	-0.92*(-1.10, -0.75)
Foreign body in other body parts	14761171 (10919497-19274891)	251.12 (187.71-812.51)	18118479 (13531105-23339852)	250.84 (186.42-45.48)	0.01 (-0.04, 0.06)

ASDR: age-standardized death rate; AAPC: average annual percentage change. The AAPCs with asterisks (*) are statistically significant (P <0.05).

Supplementary material 9. The death numbers and ASDR of different foreign bodies global in 1990 and 2019 with AAPCs over the 30 years

	1990		2019		
	Death number(95%UI)	ASDR(95%UI)	Death number(95%UI)	ASDR(95%UI)	AAPC, %(95%CI)
Pulmonary aspiration and foreign body in the airway	110044 (102406-119729)	2.13 (2.01-2.30)	108953 (100126-117329)	1.47 (1.35-1.59)	-1.25*(-1.39, -1.10)
Foreign body in other body parts	14589 (8352-19871)	0.25 (0.15-0.33)	5460 (4582-6474)	0.07 (0.06-0.09)	-4.19*(-4.37, -4.00)

ASDR: age-standardized death rate; AAPC: average annual percentage change. The AAPCs with asterisks (*) are statistically significant (P <0.05).
